# Physics and Philosophy

**Published:** 1844-04-01

**Authors:** 


### PHYSICS AND PHILOSOPHY.

I. Elements of Natural Philosophy. Being an Experimental Introduction to the Study of the Physical Sciences. By *Golding Bird*, M.D. Second Edition. London, 1844.

II. The Sources of Physical Science. Being an Introduction to the Study of Physiology through Physics; comprising the Connexion of the several Departments of Physical Science, their Dependence on the same Laws, and the Relation of the Material to the Immaterial. By *Alfred Smee*, F.R.S.

III. Caloric: its Mechanical, Chemical, and Vital Agencies in the Phenomena of Nature. By *Samuel L. Metcalfe*, M.D. Two Vol., 8vo, pp. 1100. Pickering, 1843.

The author of the first of the three works above-mentioned states, that "the best apology, that can be offered for presenting this volume to public notice, will be found in the reason which suggested its compilation, viz. the absence of any system of physics, sufficiently extended to include all those subjects with which men of education, especially members of a liberal profession like that of medicine, ought to be acquainted." The manual is chiefly intended as a text-book for the student whilst attending lectures on physics. As an additional reason for writing such a work may be mentioned the circumstance, that a knowledge of the principles of physics has been rendered imperative at the different medical boards, and now constitutes an important part of the examination, which the candidate for a diploma is called upon to undergo. He acknowledges himself considerably indebted to several French and German works for many suggestions and illustrations.

To insist on the vast importance and imperious necessity to the medical practitioner of an acquaintance with physical science, is scarcely called for in the present enlightened age. It is now universally admitted that the science of physiology rests on that of physics as its basis and firmest foundation. The skeleton is a machine in the strict sense of the term, being furnished with the various mechanical powers, as levers, hinges, &c., fitly and appropriately arranged, some to sustain pressure, others to transmit active force, some being capable of moving in one direction only, others of moving in all directions. In fact, it is in the animal body that the truest perfection and the greatest variety of mechanism are to be found. In case of dislocation or fracture of any part of the body, the question is to determine the best method of adjustment; where and how a force may be applied with the greatest mechanical advantage, and with the least risk of injury. In such a case the surgeon cannot avoid displaying, in a striking manner, either his skill or his ignorance. When under a skilful hand, with what ease does the displaced arm or thigh-bone return to its place; and, on the other hand, to what horrible torture is the victim subjected, when ignorance dares the attempt. If we pass from the osseous to the vascular system, we see that the action of this system involves hydraulic principles, whilst the organs of respiration are known to act on pneumatic principles; the *aural* and *vocal* organs are constructed on the principles of acoustics, nor can the principles on which the eye is constructed be understood without a knowledge of the science of optics. The duty of the physiologist with respect to physical science is clear enough. He should first make himself sufficiently acquainted with the physical laws, which regulate the changes incident to matter in the various forms which it may assume; he must then acquire an exact knowledge of the various forces of attraction, aggregation, repulsion, &c.; and then he will be careful to observe the phenomena exhibited in the body, and to compare them with the established laws of physics; for though organized beings have actions peculiar to themselves, and are governed by their own laws, they are still subject to the more general laws of matter. There is, in fact, scarcely a part of the animal body or an action which it performs, or an accident that can befal it, or a piece of professional assistance which can be given to it, that does not furnish illustration of some truth of natural philosophy.* Between the physical sciences and the arts of life there subsists a constant mutual interchange of good offices, and no considerable progress can be made in the one without of necessity giving rise to corresponding steps in the other. Among many other instances of this may be mentioned one in which a most familiar effect may become a safeguard of human life, and a remedy for a most serious and distressing evil. In needle-manufactories the pointers of the needles are exposed to excessively minute particles of steel, which fly from the grind-stones, and mix, though imperceptibly to the eye, as the finest dust in the air, and are inhaled with their breath; this being continued, produces a degree of pulmonary irritation, which is sure to terminate in consumption; so that persons employed in this kind of work used scarcely ever to attain the age of 40. Various efforts were made by means of gauzes, &c. to purify the air before its entrance into the lungs; the dust, however, was too fine and penetrating to be intercepted by such coarse expedients, till some ingenious person bethought him of the wonderful power of the magnet. Masks of magnetized steel are now constructed and adapted to the faces of the workmen. By these the air is not merely *strained* but *searched* in its passage through them, and each obnoxious atom is arrested and removed.†

Whilst on the subject of the importance and value of a knowledge of physics to the physiologist and pathologist, it may not be amiss to illustrate the advantage of an acquaintance with physiology to the mechanical philosopher. It is the illustrious Biot who has suggested that an attentive consideration of the animal organization, with respect to the physical laws by which it is regulated, would be found the most certain and effectual means of improving mechanical science. For instance, had the structure of the eye been attentively studied, it could scarcely have failed to lead to the invention of achromatic lenses, the eye itself being a most complete achromatic instrument, and being indebted for that quality to

* Arnott's Elements of Physics.

† Herschel's Discourse on the Study of Physical Science.

the same principle, on which the perfection of the telescope depends. In the same way, the structure of this same organ might have suggested the means of removing, or at least diminishing, the errors arising from spherical aberration.

Having now endeavoured to point out some of the more immediate and matter-of-fact advantages, which will accrue to the medical practitioner from the study of physical sciences, we cannot relinquish this part of our task without directing attention to the more remote, though not less valuable, advantages likely to result from this study. To say nothing of the liberalizing influence, which the study of the natural sciences cannot fail to exercise on the mind, we should not lose sight of the important fact that "the habits of accurate and persevering observation, of investigation, of abstraction, and of correct reasoning, are more or less produced and cultivated by the study of the philosophy of nature."* Nothing, indeed, is better calculated for cultivating that philosophic spirit which is of so much importance to the medical practitioner, than habituating the mind to the patient investigation of the various phenomena of nature, and to the close observation of those laws, by which the occurrence of such phenomena is regulated. Medicine itself being but one of the great departments of natural science, it is obvious that a mind well drilled in the general and abstract principles of the latter is much better prepared to encounter the study of the former, and much more likely to attain success in it, than when such previous and preparatory study has been neglected. We find that our eagerness to impress on the minds of medical men the urgent necessity and great importance of an acquaintance with the principles of physical science has led us somewhat away from our more immediate object, which is, to present to our readers a very succint analysis of the contents of the volume before us, and our own opinions as to its merits, and its fitness to supply the desideratum so long felt in the education of the medical practitioner by serving as a book of elementary instruction on physics.

In the Introductory Discourse we are told that the branch of Natural Philosophy which is to constitute the subject of this volume, is to consist in the investigation of the constitution of masses of matter, the laws governing them, and the mutual action of different atoms of the *same* kind, with an examination of the relation they bear to space, and to the various members of the universe. By this arrangement, the Science of Geology and of Physical Geography on the one hand, and that of Chemistry on the other, are excluded. Newton's celebrated Rules of Philosophizing are strongly recommended to the attentive consideration of the student. And here we own we were somewhat disappointed at seeing these rules called *Reguli Philosophandi,* instead of *Regulæ*—and that this is not a typographical error, or an oversight on the part of the author, is evident from its repeated occurrence. We regret to see an error of this nature disfigure a work of such pretensions—it is really a pity. The long-debated question concerning the infinite divisibility of matter is duly considered, and the idea of such infinite divisibility is shown to be absurd.

---

* See Art. Intellectual Education, in Rees's Cyclopædia.

By the way, it has more than once occurred to us that, if what is really positive in the idea of infinite divisibility be carefully considered, the thing will not appear at all so strange; when it is said that matter is infinitely divisible, the meaning is that the number of parts into which any given portion of matter may be divided is greater than any assignable number.

We shall now present our readers with a mere enumeration of the contents. The first nine chapters include the *Physics of Ponderable Matter*. Chap. I. treats of the General Properties of Atoms and Masses of Matter. II. Attractive Forces exerted between Masses. III. General Dynamics. IV. Gravitation. V. Theoretical Action of Simple Machines. VI. Hydrostatics. VII. Aerostatics or Pneumostatics. VIII. Hydro-dynamics, and Pneumo-dynamics. IX. Acoustics.—The second Part contains the *Physics of Imponderable Matter*. This part consists of Chapters, headed thus: X. Magnetism. XI. Primary Phenomena of Ordinary Electricity. XII. and XIII. Consequences of Electrical Induction. XIV. Phenomena of Atmospheric Electricity. XV. Voltaic Electricity, &c. &c.

Having now mentioned the contents of this certainly useful work, we shall take the liberty of pointing out some of what we call defects in it. As we conceive the work to be destined chiefly, though by no means exclusively, for medical readers, we regret that we do not find anything like an application of the principles of physics to the various movements and processes going on in the animal economy. Such an application would serve to illustrate those principles far better than reference to mere inanimate matter. For the purposes of such illustration a great many excellent examples may be seen in the Animal Mechanics by Sir Charles Bell.*

Another fault we have to find in this work is, that formulæ are repeatedly given throughout the work, without the student being shown how these formulæ have been obtained. Now when he has stored up in his memory a number of such formulæ, without being instructed as to the mathematical processes by which they were obtained, he has not acquired anything deserving of the name of knowledge; he believes in the truth of the formulæ, because he has confidence in the veracity of the author. We think that either these mathematical formulæ should have been omitted altogether, as they are in Dr. Arnott's Physics, or, being given, the mode of obtaining them should have been given likewise.

*Sunt delicta quibus nos ignovisse velimus.*

The faults we have pointed out, however, are not amongst the number. We dislike the affectation of science. We trust Dr. G. Bird will not misunderstand us—his book is good and without much trouble might have been made better. Every student should have gone through the various mathematical processes by which the various scientific formulæ were arrived at; in that case only will he rest satisfied of their truth; and though he may not be always able to detail, when called on, the several links of the chain, yet, having once been convinced of the truth of the Algebraic result, he knows that it must be always true—such is the great

---

* See Library of Useful Knowledge.

advantage of abstract science. He will, in fact have an habitual, though he may not have an actual, knowledge of the thing. Having, for instance, proved at any time that the focal length of a double convex, or a double concave lens, is equal to twice the product of the radii of the spherical surfaces divided by the sum of these radii, or in technical language, that it is equal to the harmonic mean between these radii, he will *know* that the same thing will be always true, though he may not be able to go through the process of proof.

---

The author of the second work mentioned at the head of this notice, viz. The Sources of Physical Science, is already favourably known by his very ingenious and clever work on Metallurgy. Mr. Smee states, in his preface, that it has been long a favourite subject with him to endeavour to investigate the physical structure of man, and to unravel the mysterious means by which all physical forces, when acting on the human frame, are converted into nervous impressions. In conducting such an enquiry, he felt it necessary to examine the sources from which the several departments of research, constituting physical science, have their origin, in order that we might be in a condition to understand the material structure of the body. For this purpose, he determined on drawing up a slight sketch of physical science, which was to form an introductory chapter to his enquiries; but, on finding that such a sketch, notwithstanding his utmost endeavours to condense it, ran out into a good-sized volume, he could not think of allowing it to form an Introduction to his Physiological Investigations; and thereupon he determined on bringing it out as a separate treatise in the form we now have it. His chief aim in preparing this work, has been to examine the mutual relations, not only of the various conditions of matter, but also of the various physical forces, the independent existence of which has been at various times assumed by mankind. The result of his enquiries has been such as to oblige him to admit but three fundamentals—matter, number, attraction,—from which, he says, under various circumstances, all physical phenomena arise. Our original intention was to amalgamate our notice of Mr. Smee's work with that of Dr. G. Bird's: but we had not been long perusing it, when we saw that the two works were *toto cœlo* different.

Mr. Smee's book is more metaphysical than physical; it is, in fact, a work completely *sui generis;* entirely unlike any other work with which we are acquainted. Anything like an analysis of a work so very abstracted, so very much removed from our ordinary modes of thinking and of reasoning, would be entirely incompatible with the well-known practical character of the Medico-Chirurgical Review, which, generally speaking, prefers to deal in things which are " sensible to feeling as to sight." Nor do we say this in disparagement of the merits of Mr. Smee's production. The book could come from no one but a man possessed of a great grasp of intellect, and great originality of mind. The first chapter of this work contains some excellent remarks on the advantages of studying abstract physics. " The right use of abstractions," says our author, " is highly convenient and important, tending to prevent parenthesis and confusion; and in fact we may say, that the capacity to employ abstract ideas in great measures gives to man his superiority over all other created things." Mr.

Locke, if we remember aright, makes the faculty of abstraction the essential difference of man. For the many recommendations of abstract science as a preparation for the study of natural philosophy, and accordingly of medicine, we would beg to refer the reader to Sir John Herschel's Discourse on the Study of Natural Philosophy, Chap. II. We must not, however, conclude our notice of Mr. Smee's book, without earnestly recommending it to the attentive perusal of those persons who may feel disposed to wing their flight into the ætherial regions of abstract essences, and to leave "dull earth behind them." Were we disposed to indulge our own feelings, we would willingly give a much more extended notice of this book; such an indulgence, however, may not be agreeable to the prevailing taste of medical men. In the appearance of such works as those of Dr. Bird and Mr. Smee we see the dawn of better days for medical science, and we feel convinced that the time will come, and is not far distant, when the preliminary education of medical men will be so improved and so extended that the occurrence of such characters as the Algebraic $x$ and $y$ in physics, or, in the application of physics to physiology, the mathematical mode of expressing the value of the moving power, as the lever, in terms of the weight and of the relative distances of the power and weight from the fulcrum, will no longer frighten the timid reader, and elicit from the more presuming, because still more ignorant one, the self-flattering remark, *si non vis intelligi, debes negligi.* We apprehend that Mr. Smee's book, as well as Dr. Bird's, stand a very fair chance of having this compliment paid them at present, and for a few years to come, not perhaps in the precise terms in which we have stated it; as ignorance of the language in which it is locked up from the class of readers now under consideration may save them so far.

---

The third work upon our list is one of considerable research. The author, in exploring the wide realm of Natural Science, has spared no labour, and has left the works of no writer, ancient or modern, unconsulted, whose lucubrations could possibly illustrate, however remotely, his theme. The reveries of Plato, the observations of Aristotle, the dicta of Hippocrates, the discoveries of Newton, the science of Bacon, the doctrines of Hunter, the theories of Davy and Liebig, of Müller and Cullen, have one and all been laid under contribution, and subjected to a searching criticism, which at least proves him to be no blind follower of authority. By the extended application of a principle in Nature, hitherto misunderstood or overlooked, he believes himself to be in a condition to enforce some of the doctrines, correct the errors, explain or expose the inconsistencies, and supply the deficiencies of these great observers. A bold generalization of this vast assemblage of facts and observations, carried out with much ingenuity of argument and felicity of illustration, leads him to deductions, which, if sound, would prove, that a much more intimate knowledge of even the most recondite operations of Nature is already possessed, than her closest observers or most rigid interrogators have ever ventured to anticipate the attainment of; while the path and scope of scientific investigation for the future become so simplified, as to consist rather in a pleasant culling of flowers and fruits in localities already indicated to us, than in the traversing with slow, hesitating, laborious step the steep hill of science. The loss of health which the sedulousness of his pursuits have entailed upon the author, would be cheaply purchased by the immortality which would infallibly attend a discovery so great; and we regret being obliged to record our conviction that no such discovery has in reality been made. Perhaps, as neither our space or time will admit of our going into the detail necessary for the purpose of confuting what we believe to be erroneous in the doctrines advanced, it is scarcely just on our parts to make this assertion. Still, having read the work with great interest, and much attention, we feel called upon to say we think its author has not made out his case. The enthusiasm which the pursuit of a favourite theory so often begets, has, amidst very much that is undoubtedly true, too often led him into the partial application of facts, an undue reliance upon the loose expressions and inexact observations of ancient authors, a misapprehension of effects for causes, an admission of properties contradictory of each other, and a far too ready assumption of his own assertions and fanciful hypotheses as sufficient make-weights against established opinions, and as stepping-stones for the most sweeping conclusions. The application of the doctrines, too, in medical practice, would lead to a most dangerously inactive and inefficient treatment of many most important diseases.

We proceed to select from the various chapters such parts as we think will best convey to the reader a general idea of the author's views.

Dr. Metcalfe regards CALORIC as the grand, if not the sole, primary agent in the various mechanical, chemical, and vital phenomena of Nature. He considers it as a material agent, and not resulting from the mere vibration or motion among the particles of ponderable matter, as represented by most modern philosophers. It is well known that all bodies are surrounded by an elastic medium, which prevents their particles from coming into actual contact with each other. This medium is caloric, as is seen by the augmentation or diminution of volume which occurs in solids, fluids, and gases, accordingly as it is added or subtracted from them. All bodies being thus full of caloric, it constitutes by far the greater proportion of the bulk of our globe, although its heating powers may, during its combination with the particles of ponderable matter, be concealed.

The following synoptical view may give some idea of the extent over which the author's investigations range.

"Having thus proved that caloric is a material agent, I now proceed to show that it is a self-active principle, capable of moving itself, and of generating motion in all other bodies. The cardinal facts which connect its agency with the general theory of physics, may be reduced to the following propositions:—

"1. That the activity or moving power of all bodies, is directly in proportion to the amount of caloric around their particles. 2. That all molecular motions, whether centripetal or centrifugal, may be resolved into the law by which caloric repels its own particles, and attracts those of ponderable matter, with forces that are inversely as the squares of the distance. 3. That the quantity of motion in the world, whether mechanical, chemical, or vital, is in proportion to the mean temperature of different latitudes, *cæteris paribus,* and diminishes from the Equator to the Poles. 4. That the centrifugal force by which planets are impelled through their orbits, is directly in proportion to the heating power of the sun. 5. That the aggregate vital energy of animals, and the development of their organization, are exactly in proportion to the amount of caloric obtained by respiration, and combined with their tissues. 6. That every variety of electricity is convertible into caloric, and the latter again into electricity; consequently they are modifications of one and the same principle. 7. That the directive power of the compass-needle diminishes from the isothermal equator to the points of lowest mean temperature, which are the magnetic poles; and that all its variations correspond with the variations of terrestrial temperature. 8. That caloric is the active principle in light, whether radiated from the sun, or generated by ordinary combustion, &c." 14.

The imparting *Motion* by caloric is illustrated in its gradually increased production among the quiescent particles of ice, as this becomes converted by caloric, first into water, then into steam. And could we suppose caloric entirely absent, then must we also suppose the system of nature totally inert. Attraction and repulsion, supposed by Newton to be ultimate principles of action, are in truth but modified effects of this same agent or primary cause. That caloric should produce these opposite effects is thus explained.

"Why then does caloric repel its own particles, and attract those of ponderable matter, with forces that vary inversely as the squares of the distance? To this primary and leading question I answer, that caloric repels its own particles because they are of the same nature, and attracts those of ponderable matter because they are of a totally different nature; that in every variety of state caloric is an essentially active principle, and incomparably more refined than any description of ponderable matter, even when expanded into the subtle form of light; for it permeates the most dense and opaque bodies, which are totally impervious to light. * * * * * * * Thus it is evident that the repulsive power of caloric, on which the volume and elastic forces of gases depend, is counteracted and diminished by an affinity of ponderable atoms for caloric; and that this attraction augments in a certain ratio, as the size of the particles increases, until it wholly predominates over the repulsive force. The atoms of hydrogen, nitrogen, oxygen, and light carburetted hydrogen, retain a large amount of caloric around them in proportion to their size; the consequence of which is, that the thermo-repulsive force greatly predominates. But when they are made to combine with each other chemically, or with carbon, phosphorus, sulphur, chlorine, &c. making gases of greater specific gravity and atomic weight, their elastic force is diminished, even in cases where little or no caloric is given out, as in the combinations of nitrogen and hydrogen, &c. &c." 109-116.

Carrying the above doctrine still farther, he arrives at the explanation of *Cohesion.*

"It still remains to prove that the cohesion of metals, rocks, and all other solid bodies, is determined by the various degrees of force with which they attract caloric; and that it is resolvable into the same cause which produces the contraction and chemical combinations of gases, or reduces them to the form of liquids. If it can be established as a general law, that the particles of all bodies are held together, with forces which vary according to the different degrees of their affinity for caloric, and that this affinity varies with every change in the relative proportions of caloric and ponderable matter, the problem of attractions will be greatly simplified; for it will be evident that cohesion is not the result of pressure, as conjectured by Newton, but that contraction and expansion, density and lightness, solidity and fluidity, are modified effects of one and the same agent; in fine, that all the operations of Nature depend on the relations of æthereal and ponderable matter.

"A thorough comprehension of the law by which caloric is chained down in a state of intimate combination with the ponderable matter of the earth, and thus prevented from expanding throughout the universe, would banish from the science of physics all those vague and absurd speculations which have been founded on hypothetical data, such as innate forces, immaterial properties, occult qualities, inertia, &c." 170.

*Gravitation*, which is usually spoken of as an universal principle of action in Nature, offers no explanation of many of her phenomena. It is, in fact, but a subordinate effect of the operation of the principle which pervades all matter. It must not therefore be considered as a cause from which the phenomena of matter proceed, but "only an expression of the general fact, that bodies tend towards each other with forces which vary inversely as the distance."

The agency of caloric in effecting *Solution* and *Chemical Attraction* is thus spoken of.

"That caloric is the universal solvent of nature is evident from the fact, that all solid bodies are convertible into liquids and gases or vapours, by a sufficiently high temperature, and reconverted into solids by its abstraction. It is equally clear that if caloric be the cause of all liquidity, vaporization and gasefaction, it must be the menstruum by which liquids and elastic fluids are enabled to dissolve other bodies.

"The most remarkable facts connected with solution, may be reduced to the following propositions. 1. That all fluids are *chemical combinations* of caloric with ponderable matter. Water is composed of oxygen, hydrogen, and caloric; sulphuric acid, of oxygen, sulphur, and caloric; and so of other liquids, the elements of which are chemically combined with it in definite proportions. 2. That no solution of a solid in a fluid ever takes place, without a transition of caloric from the solvent to the solvend. 3. That the solvent power of water and other menstruums is exalted by caloric, and diminished by its abstraction. 4. That the solutions of animal, vegetable, and mineral substances in water and other fluids, are strictly chemical combinations. It therefore follows, according to the most rigid principles of logic, that if caloric be the cause of solution, it must also be the physical cause of chemical attraction, by which salts, rocks and metals are held in a state of intimate combination with fluid menstruums." 229.

*Elective Affinity* is represented as arising from the same cause.

"All solutions and precipitations are owing to the transition of caloric from one body to another. What is termed elective affinity is owing to the stronger attraction of one body for caloric than another. By the solvent power of caloric in nitric acid, it is enabled to combine chemically with silver, the particles of which are diffused equally throughout the menstruum, making a transparent solution of nitrate of silver. But if a portion of mercury be poured into the solution, the silver is precipitated as the mercury dissolves. It therefore follows, that if the caloric of nitric acid enabled it to dissolve and combine with the silver, it must have a still stronger attraction for the particles of mercury, or it would not desert the atoms of silver for them: in short, that the silver is separated from its combination with the acid by an abstraction of caloric, for the same reason that salts dissolved in water are precipitated by a reduction of temperature." 251.

*Electricity*.—The following are the author's conclusions respecting the origin of the various species of electricity, and their identity with caloric.

1. *That the latent caloric of aqueous vapour is the true and only basis of lightning.*

Lightning is found to prevail in those regions where evaporation and rains are most copious; while, in other parts, where there is no rain (as at Lima in Peru), and in dry seasons, it does not exist—so that where there is no condensation of vapour, there is no lightning. A certain range of solar temperature is indispensable for its existence, the two being invariably connected together as cause and effect. In an uniformity of temperature, such as is secured in the tropical portions of the sea far from land, and in other parts of the world where the winds blow long in one direction, the quantity of precipitation and lightning are both greatly diminished; whereas, changes of temperature are indispensable to the condensation of atmospheric vapour, and to the evolution of its caloric in the concentrated form of electricity.

As lightning is accumulated in transparent aqueous vapour, and is immediately discharged when this is condensed, it is an optical delusion which leads to the supposition that it becomes accumulated around the surface of clouds, which, being an atmosphere of moisture, oppose its accumulation. It is its passage through these, after its discharge from the condensation of the transparent aqueous vapour in which it had been contained, that has given rise to the error.

2. *That the latent caloric of liquids is the basis of voltaic electricity in all its various forms.*

In tracing also the analogies between voltaic action and subterranean chemical forces, the above principle still holds good. The examination of the geographical situations of volcanoes proves that both their number and their power of action, are proportionate to the heating power of the sun in such localities. A farther geological examination of the surface of the earth shows "that the aggregate force by which mountains, islands, and continents, have been raised, from beneath the ocean, like all chemical transformations on the surface of our planet, is in proportion to the heating power of the sun, *cæteris paribus.*"

3. *That both are governed by the same universal law of attraction for ponderable matter, and repulsion of their own particles.*

4. That the essential properties of *positive* and *negative* electricity are the same under all circumstances.

5. That when metals are made red-hot by electricity, whether it be disengaged from a galvanic battery, from a common machine, or from a magnet, it immediately loses its peculiar power of darting through conductors and producing a shock, being *transformed into caloric,* when it excites the sensation of heat, and converts solids into liquids, vapours, and gases, which present the same properties as if generated by the action of ordinary caloric.

The second volume of the work is devoted to the consideration of the *Vital Agencies of Caloric;* and contains much matter highly interesting to the medical reader, who extends his views beyond the mere routine of the profession. After reviewing the various theories of life which have been broached, and exhibiting the more complex chemical composition of organized beings as compared with inanimate matter; the author proceeds more immediately to his proper subject with the following question:—

"Now the great question that lies at the very foundation of organic chemistry is, whether the power of forming ternary and quaternary compounds, with the aptitude for renewing their composition by assimilation and elimination, be owing to the same cause which governs the affinities of dead matter, or to some peculiar principle of a totally distinct nature, as maintained by Berzelius, Tiedmann, Müller, and nearly all the most distinguished physiologists of the present day? Nothing but an earnest appeal to Nature, and a careful examination of facts, can resolve this difficult problem." 526.

He answers it in the affirmative, and states :—

"That the power of living bodies to renew their composition by assimilation, and to reproduce their species by generation, is governed by the emphatic agency of caloric, is evident from the fact, that the power of Nature to multiply organic forms, is directly in proportion to the temperature of the earth, from the equator to the polar circles. * * * Whatever the cause may be by which organized bodies are enabled to renew their composition, must determine the actions that modify their structure and functions; for the elements of which they are formed are the same in all parts of the world, with this prominent exception, that within the tropics they are continually receiving from the sun a larger portion of that æthereal principle, (whatever men may choose to call it,) which preserves all Nature in a state of activity. The elements of the air, water, and crust of the earth, are the same in South America as at Melville Island; which is also true of all the plants and animals that inhabit the earth. The number of species, the magnitude of their forms, the complexity of organization, must therefore be regulated by the energy of the principle that causes their development, which diminishes from the hottest to the coldest parts of the globe; because in the former, the affinities of life are continually in action, but suspended for six or nine months every year, in the middle and higher latitudes."

*Animal Heat.*—As may be imagined, the author pursues this part of his subject with congenial earnestness. He advocates the chemical theory of its production, and thus speaks of the *modus operandi.*

"That a small proportion of oxygen is absorbed by the blood, and contributes to its formation, is highly probable; but I have proved that by far the greater part of it unites immediately with carbon and hydrogen in the lungs. If then it be true, that caloric is always disengaged during the formation of carbonic acid and water, and that the lungs are the source of animal temperature, the latter cannot be 'owing to the fixation or condensation of oxygen in the blood, and the combinations into which it enters in the circulation,' as maintained by Dr. Davy; nor to the 'combination of oxygen with carbon in the systemic capillaries,' as supposed by many modern physiologists. * * * * The truth is, that if carbonic acid were formed in the systemic capillaries, (as maintained by Edwards, Müller, Prout, and others,) they ought to have a higher temperature than the lungs, for the obvious reason, that whenever oxygen unites with carbon caloric is evolved, whether during ordinary combustion, fermentation, or respiration. But that the temperature of arterial blood is reduced, instead of being augmented, in the systemic capillaries, is evident from the fact, that on returning to the right side of the heart, it is found to have lost from 1° to 3° of caloric, together with its bright florid hue, and power of maintaining the actions of life, just as the steam of an engine loses its elastic force and the power of moving the piston, after undergoing a reduction of temperature.

"From all the foregoing facts we are authorized to conclude—1. That during the passage of dark venous blood through the lungs, it gives off variable proportions of carbon and hydrogen, that unite chemically with atmospheric oxygen to form carbonic acid and water, as in ordinary combustion, by which it acquires additional caloric, with a bright, florid, hue; and 2. That during its circulation through the systemic capillaries, the caloric obtained from the atmosphere is transferred to the solids, by which their temperature and vitality are maintained, when the blood returns to the right side of the heart of a dark modena hue, having lost its power of stimulating the organs, until it acquires an additional quantity of caloric from the lungs." 555.

Dr. Metcalfe animadverts at some length upon the errors into which he conceives Liebig has fallen upon this subject.

"But although Liebig has more clearly explained the chemical source of animal heat than any of his predecessors, and triumphantly refuted many absurd hypotheses; candour obliges me to say, that he has overlooked some of the most important facts connected with the theory of respiration. For example, he maintains in various parts of his late work, that atmospheric oxygen is conveyed from the lungs to every part of the body, where it unites with carbon and hydrogen to form carbonic acid and water: that 'globules of the blood, which can be shewn to take no share in the nutritive process, serve to transport the oxygen, which they give up in their passage through the capillary vessels.'"

After adducing facts in proof of the higher temperature of the blood in the left than in the right side of the heart, and the greater proportions of carbon and hydrogen in venous than arterial blood, the author continues :—

"Yet Liebig asserts, that 'arterial and venous blood have the same temperature.' It is therefore evident, that his whole theory of respiration and animal heat is fundamentally erroneous; while in some respects it is even more defective than that of Black, Lavoisier, Crawford, and Dalton, who maintained rightly, that animal heat is evolved in the lungs, and given up by the blood to the solids in the systemic capillaries; but without explaining what office it performs in any of the vital functions. And so far is it from being true, as maintained by Liebig, that 'the globules of the blood take no share in the nutritive process,' that they are far more abundant in arterial than in venous blood, as proved by the numerous experiments of Prevost and Dumas, Denis, Lecanu, and others. It is therefore manifest, that while passing through the capillaries, a portion of them are dissolved, expended in nourishing the solids, and in maintaining the various secretions.

"It is one of the most extraordinary facts in the history of modern science, that Liebig should have neglected to ascertain the difference between the temperature and chemical composition of arterial and venous blood; for this very difference constitutes the key to a right knowledge of animal physiology. Had this celebrated chemist given us more analyses and fewer hypotheses, he would have avoided many grave and fundamental errors, which now essentially detract from the value of his work." 902.

In reference to the advocates of the influence of the *Nervous System*, Dr. Metcalfe observes :—

"In reply to the arguments of Brodie, Philip, Tiedmann, Edwards, and others, who contend that animal heat is generated by nervous influence, secretion, nutrition, the condition of the blood, and muscular contraction, I shall proceed to prove that the mean healthy temperature of all animals is directly in proportion to the amount of their respiration; without which there could be no sanguification, secretion, nutrition, nervous influence, nor muscular contraction, and that they have mistaken effects for the cause of animal heat."

He then proceeds to demonstrate that the activity of all the functions, and the elevation of the temperature of the various classes of animals, is in a direct ratio with the vigour of their respiratory powers; and thus sums up:—

"We are authorised therefore, to conclude, that the intelligence of animals is directly in proportion to the development of their nervous system; but that their powers of digestion, circulation, secretion, nutrition, absorption, muscular motion, and cerebration, depend upon the amount of caloric they derive from the atmosphere by respiration. The specific office of the brain, spinal marrow, and their nerves, including those of the ganglionic system, is to endow animals with sensation, perception, memory, volition, instinct, and all the attributes of mind,—to *direct* the various movements of the body, but not to supply the moving power;—to generate ideas, but not organic products. It is very true, that the nervous system is far more highly developed in warm than in cold-blooded animals; but we shall see presently, that this is owing to the greater activity of nutrition in the former. It is also very true, that when the nerves going to a voluntary muscle are irritated, contractions are produced, and that, when divided, it can no longer contract in obedience to the commands of the will. But if the locomotive organs be not supplied with arterial blood, they become cold, insensible, and paralytic, whatever the condition of the brain may be. And if the blood be not supplied continually with caloric by respiration, it cannot excite the brain to think and will, the nerves to feel, the muscles to contract, and the glands to secrete." 600.

*The Action of the Heart* is excited by the blood which its cavities receive; and this fluid is enabled to effect this by reason of the caloric it has derived from the lungs. This is seen in the experiment of causing the renewal or suspension of the pulsation of the organ, when it has been removed from the body, accordingly as it is exposed to an elevated or diminished temperature, and that independently of the influence of oxygen or other agent. So, too, corresponding to the amount of caloric they receive from their vigorous respiration, the pulsation of the heart is stronger and more frequent in birds than in any other animals; while whatever tends to lower the temperature of the body also diminishes the action of the heart.

*Digestion.*—The solvent power of the gastric fluid does not depend upon the influence of the nerves going to the stomach, as their division does not destroy it.

"Nor have we ever had the slightest proof that electricity is essential to the process; for the experiments of Dr. Philip have been repeatedly proved to be fallacious, and therefore require no comment. But that caloric is the principal agent upon which the solvent power of gastric juice depends, would appear from a variety of considerations. 1. We have seen that digestion is far more rapid in birds than in mammalia, and more rapid in the latter than in cold-blooded animals. 2. It has been proved by the experiments of Dr. Beaumont, that when gastric juice is taken from the stomach, and kept in phials at the temperature of the body, it converts aliment into chyme; but that when kept below the temperature of 40°, it produces no more change than so much water. 3. That the digestive power in man is in proportion to the quantity of his respiration, which is augmented by moderate exercise and agreeable emotions, but diminished by repose and the depressing passions. 4. I also found that when the stomach was weak, digestion was wholly arrested by drinking half a pint of cold milk during winter, attended with flatulence, colic and nausea, that lasted for above twelve hours. The danger of drinking cold water when exhausted by over-exertion, is well known in warm climates, where it often destroys life suddenly. Dr. Beaumont states, that on giving to St. Martin a gill of water at 55° when the stomach was empty, its temperature was reduced from 99° to 70°, at which it stood for a few minutes, and did not regain its normal standard until thirty minutes had elapsed. Cholera, gastritis, dysentery and diarrhœa, are often produced by cold drinks, ice-creams, &c." 629.

*Process of Nutrition.*—This the author is by no means disposed to look upon as the great mystery supposed by some physiologists. After repeating the changes in temperature and composition which the blood undergoes during its circulation, he continues:

"In this state, it excites the left ventricle of the heart to contract, by which it is impelled into the ultimate tissues of the whole body, where the caloric just received in the lungs, together with a portion of arterial blood, percolate the delicate coats of the capillaries, unite with the solids by vital affinity, and form the various secretions by glandular action. As proofs of what has just been stated, the temperature, vital activity, and renovation of all the organs, are constantly maintained at the expense of arterial blood, during its conversion into the venous state, while passing through the systemic capillaries, where its temperature is reduced, the number of its organic particles diminished, and its power of sustaining the healthy state of the different functions is greatly impaired,—when it returns to the lungs for a fresh supply of organic particles and living fire. If the temperature of the blood were not raised above that of the solids while passing through the lungs, there could be no transition of caloric from one to the other—no combination of its proximate constituents with the membranous, bony, muscular, and nervous tissues. Nor could chyle and venous blood be converted into the arterial state, without giving off carbon and hydrogen in the lungs, by which caloric is evolved, the temperature of the blood elevated, and its composition renewed. * * * * * * The inference is obvious that animal heat is the agent by which the vital affinities are produced, and the activity of the various tissues maintained. Nor is this any more remarkable, than that the chemical union of water with the aromatic constituents of tea, coffee, medicinal infusions, and many other bodies, including the various salts, should be owing to the transition of caloric from one to the other. Nor is it more strange, that the caloric disengaged in the lungs during respiration, should there convert chyle into blood, than that the same active principle should cause oxygen and hydrogen to unite in the formation of water, or that solar caloric should determine all the chemical and vital transformations of the vegetable world." 666.

After protesting against the hypothesis which explains secretion and nutrition by nervous influence, he continues—

"Is it then possible that physiologists are in doubt whether the fluidity of the blood is owing to the same cause which determines the fluidity of the ocean? Or is it possible that the Author of Nature would have ordained that the temperature of arterial should be higher than that of venous blood, if the difference were not essential to the well-being of the animal economy. The truth is, that this 'slight difference' (as it is termed by Dr. Southwood Smith) constitutes one of the most important facts connected with the whole theory of physiology, pathology, and therapeutics. In accordance with the views of the ancients, who believed with Homer, that 'strength is derived from spirits and from blood,'—it is now completely established, that the contractile power of the muscular system, whether voluntary or involuntary, is directly in proportion to the quantity of arterial blood with which they are supplied, and the rapidity of the circulation through them. But that caloric is the spirit on which its power of maintaining their activity and strength depends, is manifest from the facts already stated, that if the temperature of the blood be not raised above that of the solids, while passing through the lungs where it is formed, it cannot unite with their substance: and that whenever the general system is reduced below the natural standard by the abstraction of its vital heat, or by a deficient supply of it by respiration, all the energies of the animal machine are proportionally diminished.

"On the other hand, whenever the temperature of the living body is raised much above the natural standard, so that the solids are brought nearly to an equilibrium with that of the arterial blood, the operations of secretion and nutrition are nearly suspended, and all the energies of life, proportionally diminished. The reason of which is, that under such circumstances, there is very little caloric transferred to the solids in combination with the blood, which therefore returns to the right side of the heart, of nearly the same temperature, florid hue, and quantity of organic particles, as when it left the lungs. Hence it is, that when the system is raised above the natural standard, as during the immersion in the hot bath, or surrounded by an atmosphere from five to ten or more degrees above the healthy temperature of the body, as in the tropical portions of Africa, New Holland, India, and South America, during the heat of the day, all the powers of mind and body are prostrated, attended with syncope, apoplexy, and frequently death, if not relieved by cooling ablutions, by which the system is reduced to its natural *plus* and *minus* condition of arterial blood and of the solids. For it is a certain fact, that without this essential condition, none of the vital functions could be carried on." 672.

*Climate.*—Having thus stated the importance of the agency of caloric in the organization of animated beings, the author next illustrates his theory by a long and interesting review of the effects which it produces upon the physical, intellectual, and moral condition of man, as evidenced in the inhabitants of different climates. He observes—

"Thus it is, that no great, wealthy, populous, and highly civilized nation, has ever existed in either very hot or very cold climates. If man be dwarfed in stature, and all his higher faculties blunted, by the torpifying agency of intense and long-continued frost—he is equally degraded by the perpetual influence of an elevated temperature, which blackens his skin, renders his hair coarse, harsh, and curly, while it impairs the vigour and beauty of his whole organization."

After describing the defective organization of the Negro, he continues:

"It is therefore not surprising that for thousands of years the natives of Africa have remained in a state of the grossest barbarism—nor that they have been reduced to slavery by the Egyptians, Phœnicians, Persians, Greeks, Romans, and nearly all the nations of modern Europe.

"It is certainly true, that some of the most populous, wealthy, and highly civilized nations of antiquity, flourished in Southern Asia and Northern Africa; as in India, China, Arabia, Syria, Egypt, Tyre, Sidon, and Carthage. But the climate of these countries is moderate, compared with that of tropical Africa and New Holland; or intermediate between what I have denominated hot and temperate climates. For example, the mean annual temperature is 70° at Algiers; in Winter it is 61.4° and 82.8° in Summer; while at Cairo in Egypt, the mean of the year is 72.4°, that of Winter 58.4°, and that of Summer 85.8° Corresponding with this intermediate climate, is the physical character of the natives, who are neither black nor white, but dark brown, olive, or yellow; with long and slightly curled hair, regular features, sparkling eyes, slight but muscular frames, well-formed heads, and a considerable share of intelligence.

"It has been said that 'there is nothing more difficult to explain fully, than the immense superiority of Europe over the other quarters of the world.' But I shall endeavour to show that it is, because the climate of middle and southern Europe is more temperate and uniform. In the first place, there is nothing better established by history, than the superiority of man in temperate latitudes. The once populous and wealthy nations of Egypt and Syria were conquered by the Persians, Medes, Greeks, and Romans—the Chinese by the Tartars—Italy by the Germans, Goths, and Huns—North Africa by the Romans—and the East Indies by the British, who are now invading China.

"The climate most favourable to a high development of the human race, would seem to be one in which the mean annual temperature approximates that of the whole earth, which is about 50°, and in which the variations are small, as in the middle latitudes of Europe, where the average of Summer is seldom more than 10° above the mean of the year, and that of Winter seldom more than 10° below the same standard. For man has attained to greater perfection in Great Britain, Germany, Holland, Belgium, Prussia, Sweden, and Russia, than in any other quarter of the world." 721.

In considering the varieties of stature, configuration, &c. which the inhabitants of various countries present, we must ever bear in mind a "law of Nature, *that in man and all the higher animals, respiration is augmented in proportion as the surrounding temperature falls below the point at which they are capable of maintaining themselves at the natural standard, but diminished in proportion as the atmosphere rises above that point, cæteris paribus*." Thus, in the inhabitants of some of the coldest regions, the thorax becomes disproportionally large to the rest of the body; and yet the organization is not vigorous in proportion to the quantity of caloric passing through the tissues, owing to its rapid abstraction by the surrounding cold air. In tropical countries the chest is more narrow, and less in circumference than in higher latitudes—the caloric of respiration, not being so readily dissipated, accumulates in the system, "producing a disagreeable sensation of preternatural warmth, the tendency of which is to diminish the action of the lungs."

The ill effects of the climate of Africa are spoken of as follows:

"The greater mortality of tropical Africa than of the West Indies must also be sought in the vast difference between their climates. For although the mean annual temperature is nearly the same, it rises 20° higher during the heat of the day in Central Africa, while it often descends to 60° at night, and sometimes to 50° or even to 42° in the morning before sun-rise—making a diurnal range of from 50° to 70°; whereas in the West India Islands it is only from 10° to 20°. Besides, owing to the vast bodies of alluvial lands, which are converted into swamps and morasses, by periodical inundations of the rivers in tropical Africa, a much larger amount of malaria is generated, than in islands of a moderate size and a milder temperature.

"Corresponding with this state of things, we learn from travellers that the negroes on the Senegal, Gambia and Niger, like those of Central Africa, are generally a feeble, indolent, and phlegmatic race, who seldom live beyond 60 years of age—who are grey, wrinkled and decrepid with age at 45; while in health, strength, beauty, and intelligence, they are greatly inferior to the natives of the elevated plains of ancient Æthiopia and Abyssinia, or those of North or South Africa. But when removed to the milder climate of St. Domingo, and other West India Islands, it is said by Collins and others, that, in two or three generations, they improve greatly in all the endowments of body and mind. As a proof of this, some of the black Creoles of Hayti have been distinguished for courage, ability, information, and patriotism. We have also seen in the temperate climate of the United States, the increase of the negro population is nearly the same as that of the whites, notwithstanding the number of emigrants annually added to the latter.

"It is, therefore, not true, as supposed by Prichard that negroes are under the same disability to thrive and multiply in cold and temperate climates, as Europeans within the Tropics. Nor is it true, as maintained by Caldwell, that 'the negro is most healthy, long-lived, and attains the highest perfection of his nature in his native country;' on the contrary, there is but little reason to hope, from the history of the past, that he will ever rise much above the state of barbarism in the tropical portions of his own country. Why, then, should men, calling themselves philanthropists, be so anxious to remove the black population of the United States, (where they multiply faster, and live longer than almost any other people in the world,) to the Western Coast of Africa, where the mean duration of life is not much above 20 years, as among the colonised blacks of Sierra Leone and Liberia? It is a wicked and reckless expenditure of life and money." 774.

The effect of climate, in determining the diseases of man, is thus spoken of—

"Thus we perceive that in Britain, the Canadas, Nova Scotia, and New Brunswick, about one-half of the mortality is from diseases of the respiratory organs; whereas, in the warm climate of the Mediterranean, they form about $\frac{1}{5}$ of the whole,—in the West Indies nearly $\frac{1}{7}$,—and not $\frac{1}{100}$ part among the white troops in tropical Africa. We are also informed by Tulloch, that in the East Indies, where the mean annual mortality varies from 70 to 90 per 1000, nearly the whole was from fever, dysentery, diarrhœa, and diseases of the liver:—that, among 74,850 native troops serving in Madras, the mean annual ratio was only 1 per 1,000 from all diseases of the lungs: and but 2·4 in the Mauritius and at St. Helena. We farther learn from Dr. James Johnson's work on Diseases of Tropical Climates, that from 1827 to 1836, the proportion of deaths from diseases of the respiratory organs was $\frac{1}{11}$ of the whole at Calcutta, at Chinsurah $\frac{1}{16}$, and at Berhampore $\frac{1}{20}$. In North Africa, the mortality from phthisis is still less, according to M. Guyon, a medical officer of the French army, who states that, from 1838 to 1841, it was only $\frac{1}{40}$ of the whole among the Moors at Algiers, among the Jews $\frac{1}{37}$, and about $\frac{1}{20}$ among the Europeans.

"But why is it, that the mortality from diseases of the lungs is so much greater among the negroes of the West Indies than among Europeans? And why are the latter so much more liable to fevers when removed to tropical climates, than the natives. The solution of these difficulties must be sought in the radical differences of organization of men, and other animals in cold or temperate, and in hot climates. For example, we have seen that, owing to the high temperature of tropical Africa, for the greater part of the year, during the heat of the day, respiration is proportionally diminished, and the lungs exercised less, than in colder climates, by which the size of the thorax is accommodated to the wants of the system. So that when removed to the West Indies, where the maximum temperature is from 10° to 20° lower, the natives of Africa are unable to obtain caloric from the atmosphere by respiration, as fast as it is abstracted by the surrounding media, especially in the high lands, or during the prevalence of northerly winds, and early in the morning, when the air is damp.

"The consequence is, that, under such circumstances, they are often found shivering with cold, but never complain of the most intense heat of the sun, which is no less delightful to their feelings than conducive to health. * * * * Nor is it until several generations after his removal to a colder climate, that the thorax of the African, is developed to the same extent as that of the European; so that, like the monkey, the lion, tiger, and leopard, he is proportionally subject to diseases of the lungs. On the other hand, as the lungs are more exercised in temperate and cold climates, the thorax is more highly developed among the whites, who therefore obtain a larger amount of caloric by respiration, *cæteris paribus*, by which they are enabled better to resist the influence of a low temperature. But, for this very reason, when removed to the burning climate of tropical Africa, India, and America, they receive caloric from the atmosphere by respiration faster than it is carried off, causing the temperature of the body to rise above the natural standard, and predisposing it to attacks of malignant fever." 784.

Why the respiratory organs should be so especially liable to become diseased in the higher latitudes is thus stated—

"I answer, because the pulmonary air-cells and Schneiderian membrane, present an extensive surface to the atmosphere; and that, in cold climates, they are exposed to the paralysing influence of a low temperature, more constantly than any other part of the body. The consequence is, that as animal heat is the immediate cause of all vital action, the chemical function of the lungs, sanguification, and circulation, are diminished, constituting the first link in the chain of the morbid phenomena, which are essentially the same in all cases, modified, however, by the condition of the system, the nature of the part more immediately affected, and the greater or less intensity of the exciting cause."

According to the condition of the individual, and the extent of exposure, mere catarrh, any of the inflammatory affections of the lungs, or phthisis itself may be induced. The author believes, indeed, that *phthisis* may, in almost every case, be traced to inflammatory affections of the lungs or bronchi, or to the influence of repeated colds which might often have been avoided. Indeed, he seems to doubt the hereditary nature of the disease, although admitting that it may be induced, even at the earliest ages, by exposure to predisposing and exciting causes.

However formidable the influence of cold may be in the production of disease, a long-continued exposure to a very elevated temperature is still more fatal to the human race:—thus, the yellow fever of the East and West Indies, the malignant African Remittents, &c. are frequently of the most deadly character. In such climates "the process of respiration is nearly arrested, and the temperature of the solids is raised so nearly to an equilibrium with that of the arterial blood, that the transition of caloric from one to the other, on which the process of nutrition depends, is nearly suspended, and all the energies of life are proportionally diminished."

"*Malaria*.—Although we may not know precisely whether malaria depends chiefly on the agency of carbonic acid and vicissitudes of temperature, or some yet undiscovered effluvia, in conjunction with heat, cold, and moisture, we can clearly distinguish the manner in which they operate, and the effects they produce on respiration, sanguification, secretion, nutrition, and all the vital functions. Whether generated in large cities, confined dwellings, or marshy districts, its morbid influence is essentially the same, modified, however, in all cases, by climate and season, being always more concentrated and malignant where the temperature is highest *cæteris paribus*. And whether cholera be contagious or not, like typhus, yellow fever, and plague, it is generated by foul air, bad diet, filth, cold, moisture, fatigue, and the depressing passions, where none of them before existed." 821.

Of investigations into the nature of malaria, the author thus speaks—

"I therefore appeal to the candour of all enlightened minds, whether it is not more in the spirit of true science, to ascertain the influence of carbonic acid, and of all other gaseous emanations which are known to arise from the decomposition of dead matter, than to seek for the nature of malaria in some mysterious and hypothetical condition of the atmosphere about which nothing is known? And whether vicissitudes of temperature do not constitute a still more important condition of what is termed malaria, than even carbonic acid, or any other mephitic gas. For it is certain, that in hot climates and seasons, exposure to fatigue during the heat of the day, and to the cool damp air of night, are by far the most common exciting causes of fever, the malignity of which is in proportion to the elevation of temperature where it prevails, *cæteris paribus*." 827.

The last division of this work is principally devoted to the application of the principles already advanced to hygiene and therapeutics, and to a new theory of fever.

*Exercise.*—Lavoisier instituted the first accurate experiments upon the influence which exercise exerts upon respiration.

"Thus it would appear that respiration is augmented above 100 per cent. by exercise, beyond what it is in a state of repose; and that it is increased about 40 per cent. after taking a hearty meal. Hence men in health can endure a temperature of 32° during moderate exercise, with more comfort and safety, than one of 50° while in a state of rest; and that, when supplied with an abundance of food, they can endure the most intense degrees of cold, so much better than during abstinence, as observed by Franklin, Ross, and other travellers in the Arctic regions.

"As it is a law of nature that the cause of force is always expended in producing motion, it will be found that the vital heat of animals is wasted more rapidly during violent exercise, than it is obtained by respiration. The truth of this proposition is proved by the well-known fact, that long-continued muscular exertion is followed by more or less exhaustion, and by diminished power of enduring cold. For notwithstanding the elevation of temperature thus induced, the absolute amount of caloric in a state of combination with the organs, is reduced below the usual standard; while it is equally obvious, that in proportion as respiration is increased during violent exercise, must it be diminished afterwards, for the plain reason, that a large amount of carbon and hydrogen by which the vital combustion is supported, has been already given off in the lungs.

"As a further proof that the caloric obtained by respiration, and transferred to the different organs in combination with arterial blood, is forced out and expended during their action, it was ascertained by Dr. Granville, that during the violent contractions of the uterus which mark the progress of difficult parturition, its temperature sometimes rises to 110° or even 120°. Edwards also relates a case of tetanus, on the authority of Dr. Prevost, in which the temperature of the body rose 12.6° during the spasms." 879.

The effects of exercise carried to the extent of *fatigue*, are thus spoken of:—

"A correct knowledge of the manner in which animal heat is expended by exercise, will enable us to explain why it is that, when greatly fatigued, health is often destroyed by immersion in the cold bath, which suddenly reduces the body below the natural standard, paralyses the lungs, diminishes respiration, and thus lays the foundation of pneumonia, phthisis, or some other fatal malady, if not prevented by immediate recourse to the warm bath, or the application of dry heat, until the circulation is perfectly restored. Nor is there a more frequent predisposing cause of fever, dysentery, cholera, diarrhœa, and congestion of the liver, than exposure to rain, fogs, damp night air, or even a moderately cool draught of air, when fatigued by over-exertion, especially in warm climates, where the small amount of caloric obtained by respiration is much sooner expended by exercise than in the higher latitudes; so that a very slight exposure brings on a chill, and torpor of the internal organs.

"Many persons imagine—and I am not sure that even medical men are wholly free from the same error—that the danger from exposure to cold after fatigue, is owing to what they call an *over-heated* state of the body. But there is not the slightest danger of taking cold when the body is over-heated by the warm-bath, as proved by the Russians, who have found that it enables them to endure cold for a much longer time than they otherwise could; and that, after coming out of the vapour bath, the lower orders often roll themselves in the snow with impunity. The fact is, that whenever the circulation is languid, respiration is augmented by the warm bath, which increases the action of the heart, and causes a larger amount of blood to pass through the lungs in a given time." 886.

*Aliments.*—The following are the author's conclusions:—

"1. That each Zone affords in the greatest abundance those descriptions of aliment best suited to maintain the well-being of its inhabitants. 2. That excessively cold climates abound with animals which contain a large amount of oil and fat, that are rich in carbon and hydrogen, which afford an abundant supply of animal heat where it is most required. 3. That the middle latitudes abound with grass, grain, and domestic animals, which are less numerous, and contain a much smaller proportion of fat in hot climates, where there is an exhaustless profusion of saccharine fruits, gums and farinaceous aliments, that afford less carbon and hydrogen, therefore, less caloric by respiration, than animal food. 4. That the various species of grain afford a much larger amount of actual nourishment, than an equal weight of animal food, if we except cheese, butter, fat, and lean meat deprived of water. 5. That, during the process of respiration, starch, sugar, gum, and fat are converted into blood, by absorbing nitrogen from the air, and by giving off carbon and hydrogen; consequently, that the elements of respiration, when combined in due proportion, are employed in nourishing the solids, like fibrin, albumen, and casein, of both animal and vegetable food. 6. That as the chemical composition of all animals is the same, herbivora must derive a portion of their nitrogen from the atmosphere, because their food does not contain enough of that element to maintain their nutrition and growth, which are even more rapid than in carnivora. 7. That the living body is a self-repairing machine, which has the power of transforming both ternary and quarternary compounds into its own tissues; and when wholly deprived of food, is capable of living many days on its own ruins, which are repeatedly renovated in the lungs, where they are also converted into carbonic acid, water, and other inorganic compounds. 8. That the rapid increase in the weight of the body after long abstinence or illness, the speedy healing of broken bones, the filling up of ulcers, and the rapid growth of the herbivora, tend to prove, that the nitrogenized portions alone of vegetable food are insufficient to account for the renovation of their composition, and the supply of waste. 9. That a suitable variety of vegetable aliments is better adapted to the organization, health, strength, intelligence, and moral excellence of the human race, than a diet of animal food alone. 10. That although spirits, wines, and malt liquors, when taken in moderation, elevate the temperature of the body, augment the circulation, produce a temporary flow of spirits, remove the sensation of hunger, fatigue, and other disagreeable feelings—they impair the vital properties of the blood, and diminish its coagulating power when used in excess—derange the nutritive process, cause a dropsical or phlegmatic condition of the solids, and gradually destroy the *vis medicatrix naturæ*, as shewn by the slow healing of wounds and ulcers in intemperate drinkers." 937.

*Sleep.*—The author believes that the system increases its dimensions during sleep, and that these become diminished during action, or, to use his own phraseology, that, during exercise the expenditure exceeds the income, while, during sleep, the income exceeds the expenditure. If the body be well covered, more animal heat is retained within it than during exertion; but as less is now obtained by respiration, if it be not covered it becomes chilled. Whatever diminishes the circulation of arterial blood through the brain, or impairs the vital activity of this organ, produces a tendency to sleep. Thus, full meals, spirituous drinks at night, &c. determine a flow of blood from the brain to the stomach; as do heated rooms, &c. to the surface; and, in both cases, sleepiness results. Hence, too, the delightful and soporific influence of the warm bath, after great fatigue.

"But excessive warmth is unfavourable to sound sleep—1st, by increasing the circulation of blood through the brain; and, 2d, by raising the temperature of the solids nearly to an equilibrium with that of the arterial blood; by which the combination of its particles with the solids is diminished. Hence it is, that when oppressed with too much covering, we feel languid and unrefreshed on rising in the morning; and that men sleep more soundly in temperate than in hot climates, where nothing more conduces to healthful repose than cooling ablutions, or the tepid bath, before going to bed.

"Whatever greatly diminishes the nutritive process, tends to prevent natural sleep, which is therefore always imperfect, if not wholly interrupted during fever, and many other forms of disease. It is also frequently prevented by over activity of the nervous system, caused by mental anxiety and too intense thinking, which interfere with the nutritive process, and induce a feverish state of the brain, that may be relieved by cold applications to the head, putting the feet into warm water, and then getting into a warm bed—all of which tend to equalize the circulation, and diminish the morbid activity of the brain.

"It is maintained by Dr. Billing, that a warm bed is favourable to sleep, by causing a *plethoric state of the brain.* But, according to the observation of Blumenbach, the circulation is diminished in the brain, and its vessels are less turgid during sleep than when we are awake. Hence, it is, that strong tea and coffee prevent sleep, by augmenting the circulation and activity of the brain; and that when the blood is determined from the surface and extremities by sleeping in a cold bed, and sent to the brain in augmented quantities, we are kept awake until the circulation is equalized by warmth. Sleep is more sound during the early part of the night, when the nutritive process is actively employed in reparing the previous waste, than towards morning, when it is often partially interrupted by dreams." 949.

*On Temperaments.*—Dr. Metcalfe attempts a simplification of the classification of these. Since caloric is the agent by which the blood is formed and converted into the various tissues, it must be the ultimate regulator of constitution and temperament. As the quantities of the organic particles of the blood, and the vital energy of animals are proportionate to the capacity of the lungs and amount of respiration, so a broad, deep, full chest becomes the mark of what the author calls the *dynamic temperament;*

while persons whose chests are narrow, flat, and small, and in whom respiration, sanguification, and nutrition are imperfectly performed, belong to the *adynamic temperament.*

The dynamic embraces the sanguine and choleric of Richerand, and the thoracic of Thomas, with some other subordinate varieties. Thus, in persons of large chest, sound lungs, robust health, and small brain, we have what may be called the *muscular* or *athletic* temperament, as in boxers, pedestrians, various savage tribes, and some classes of operatives. With a broad, round, full, chest, and muscles moderately developed, there may be a large and well-formed brain, constituting the *cerebral* or *intellectual* temperament—the choleric of the ancients—comprehending some of the noblest specimens of the human race. When the chylopoietic viscera are more highly developed than the brain, we have what Thomas terms the *abdominal* temperament—Shakespeare's " fat ribs and lean pates." The thorax may be well formed, and the head moderately large, but an undue proportion of the rich arterial blood is expended in the secretion of fat—such excessive secretion denoting imperfect sanguification produced by excessive alimentation, want of exercise, or some defect in the function of respiration. All these varieties of the dynamic temperament may be so mixed in different individuals, as to form several subordinate varieties.

" On the other hand, whenever the thorax is below the ordinary size, or the function of respiration is imperfect, there is a deficiency of animal heat, and of rich arterial blood, with a languid state of all the vital forces—constituting the weak or *adynamic* temperament—whether the muscles, brain, or abdominal organs predominate. But if the nervous system be considerably more developed than any of the other tissues, we have that variety termed by the ancients the *melancholic,* which corresponds with the *nervous* temperament of modern physiologists. For they are both represented as marked by a small chest, pale, sallow, or livid complexion, (indicating a deficiency of bright arterial blood,) a languid circulation, torpor of the stomach, bowels, and of all the secretions, a spare habit, soft, small, and feeble muscles, morbid sensibility, sudden fluctuations of temper, with a predisposition to nervous and spasmodic affections. And we have already seen, that the principal difference between this temperament and the phlegmatic is, that in the latter the abdominal organs are more highly developed than the brain, which is weak and lethargic.

" It is therefore evident that the adynamic temperament, whether cold and dry, or, cold and moist—whether denominated melancholic, nervous, or phlegmatic, and whether hereditary or acquired, is rather the effect of disease than a primitive or natural constitution. And that it is often acquired, would appear from the well-known fact, that many distinguished individuals who were originally of the sanguine temperament, have been rendered melancholic or nervous by grief, anxiety, intense study, a sedentary life, and repeated shocks of adversity; as exemplified in the characters of Dante, Petrarch, Tasso, Pascal, Cromwell, Newton, Voltaire, Rousseau, Zimmerman, Collins, Cowper, Burns, and Byron. The misery of such men is owing to a greater activity of the nervous system, than it has the physical power of supporting." 1004.

Although as a general rule it may be truly stated that the intellectual power of man is proportionate to the size of his brain, and especially the anterior portion of it, yet this is not always the case.

" Why is it that many individuals, with moderately-sized heads, possess far greater power and activity of mind than others who have large and well-formed heads?

"In reply to the above, I have already shewn it to be a law of nature, that the power of any organ is in proportion to the amount of rich arterial blood with which it is supplied, the quantity of caloric that passes through it in a given time, *cæteris paribus*, and depends upon the amount of respiration.

"It is a great blessing to be born with a large thorax; for it offers the surest pledge of vigorous health and long life. Had all men such chests as the Duke of Wellington and Daniel O'Connell, disease would be greatly diminished, and the duration of life augmented. I am informed that six individuals of the Wellesley family, recently alive, had arrived at the aggregate age of 443 years, making the average of each 74 years. And Mr. O'Connell stated, in a speech last year, that among 22 children of his grandmother, 11 arrived at the age of 96 and upwards. Like the heroes of Greece and Rome, the physical energies of these illustrious Irishmen were developed by exercise in the open air, and its free circulation through their capacious lungs, without which they never could have endured so much bodily and mental exertion. With a full chest and sound lungs, men are enabled to endure degrees of cold, muscular exercise, loss of sleep, excesses in eating and drinking, that would soon shorten the lives of ordinary individuals." 1007.

*Theory of Spasmodic Diseases.*—That these are produced by reason of a diminished vitality of the brain, the author believes, because—

1. That in all the higher orders of animals convulsions are invariably produced by a great and sudden loss of blood; and may be caused by the sudden abstraction of animal heat, independently of the loss of blood, as in cramp from swimming in cold water. 2. That convulsions and death follow the introduction of active poisons into the stomach or circulation. 3. That a reduction of temperature several degrees below the standard is attended, as in the cold stage of ague, with a constricted state of the skin, and a spasmodic condition of the whole system. 4. Convulsions are produced by strangulation, or by whatever arrests or greatly diminishes the process of respiration, whether it be exposure to the mephitic gases, the rarefied air of high mountains, a blow on the head, or violent emotions of terror and other depressing passions.

On the other hand, no spasmodic disease can exist while the brain and general system are supplied with an abundance of good arterial blood.

The author applies these principles to the explanation of the chief spasmodic diseases, as epilepsy, tetanus, hydrophobia, &c. At the commencement, or in the progress of all these, he sees as their cause a diminution of the vital powers produced by the imperfect circulation of a deficiently arterialized blood. His means of relief consists in applying sufficiently early the warm water or vapour baths; and he criticizes with severity the inconsistent nature of many of the heroic remedies so frequently essayed.

*Theory of Fever.*—The author, having alluded to the fact that the former portions of his work prove, that the predisposing and exciting causes of disease tend to the reduction of the temperature of the body, declares, "There never was a general fever without a previous reduction of temperature, which is the first prominent link in the chain of morbid phenomena, and the invariable cause of all the following symptoms." During such chill, the respiration is diminished, and the heart's action lessened in force and frequency. The vital properties of the blood become impaired during the defective respiration, and from the absence of due elimination of its excretory portions.

"It is therefore obvious that the proximate cause of fever is not an excess of bile, as maintained for the last two thousand years; nor debility of the brain and a spasmodic state of the extreme vessels, as supposed by Cullen; nor inflammation of the stomach and bowels as supposed by Broussais; but that the primary cause of these and all the other symptoms is a loss or deficient supply of the animating principle, and a consequent vitiated state of the blood, to which may be traced the universal debility of the brain, bowels, voluntary muscles, and the general feeling of soreness, with an aching in the back and limbs."

Nevertheless, during this, the cold stage, respiration, though imperfect, still goes on, and the caloric, which should have been employed in the process of nutrition, gradually accumulates; and the action of the heart becoming proportionally vigorous, the condition of torpor and cold becomes exchanged for one of inordinate heat and activity. The additional heat now obtained by respiration is, however, incapable of performing any healthy vital office, until the blood be restored to its natural state. In the case of intermittents, such purification occurs during the sweating stage. All varieties of fever are but modifications of the same disease, arising from different degrees in the intensity or duration of the causes producing them—the extent to which the vital properties of the blood become impaired, determining either a mere quartan intermittent, or the most deadly form of typhus, yellow fever, &c.

The author, believing caloric to be the grand animating principle of life, conceives almost all hygiene to consist in maintaining its source, the respiratory functions, in due activity; and in avoiding its direct abstraction by damp, cold, and excessive exertion. So, too, his treatment of fever, when it does occur, is simple enough; for, eschewing all heroic remedies, nay medicines of any activity, altogether, he finds his panacea in the application of heat during the cold stage, and in its abstraction during the hot.

*Theory of Inflammation.*—Inflammation and fever are modifications of the same radical disease. The immediate cause of capillary circulation resides in the blood, and is owing to the transition of caloric from the blood to the solids, as in the process by which various fluids are forced through the pores and small tubes of dead matter. "In the great majority of cases an attack of inflammation is brought on by the immediate influence of cold, which retards the circulation through the capillaries, and diminishes their contractility. The consequence is, that they are dilated by means of the *vis a tergo*, engorged with blood, and tumefaction is induced. In the mean time, owing to the weakness and diminished cohesion of the vessels, there is an effusion of serum, lymph, and sometimes of red blood, into the cellular tissue, or other surrounding parts, by which the swelling is still farther increased. And as the onward motion of the blood is impeded, it is prevented from receiving the vitalizing influence of respiration, by which its nutritive powers are impaired: so that the animal heat sent to the part in combination with arterial blood, is not properly united with the solids, as during health, but given out in the free state, causing a local fever. Hence the redness, tension, swelling, and heat, which are attended with more or less pain, owing partly to compression of the nerves, partly to morbid sensibility produced by the preternatural temperature, and still more, perhaps, to a failure of the nutritive process, any derangement of which is always accompanied with disagreeable sensations."

In treating inflammation we should endeavour at an early stage to remove its proximate cause, by increasing the circulation through the capillaries, which is best accomplished by the use of warm applications; and where the action of the heart is such that more blood is forced into the weakened vessels than can be circulated through them, or where extreme plethora prevails, moderate bleeding, general or local, may be had recourse to. "But the leading object should aways be to restore the action of the weakened vessels, by a judicious and varied application of the agent on which all the powers of life depend."

Although we cannot agree with the author in attributing so universal an agency to the operation of caloric, we can recommend his work to the perusal of our readers, not only on account of the ability with which the theory it advocates is developed; but because it contains a very good *resumé* of the facts of medical and collateral science bearing upon the subject.

